# High mobility group box 1 gene polymorphism is associated with the risk of postoperative atrial fibrillation after coronary artery bypass surgery

**DOI:** 10.1186/s13019-015-0301-2

**Published:** 2015-06-25

**Authors:** Can Qu, Xiao-Wen Wang, Chun Huang, Feng Qiu, Xiao-Yong Xiang, Zhi-Qian Lu

**Affiliations:** 1 Department of Pharmacy, The First Affiliated Hospital of Chongqing Medical University, Chongqing, 400016 People's Republic of China; 2 Department of Cardiothoracic Surgery, Shanghai Jiao Tong University Affiliated Sixth People’s Hospital, Shanghai, 200233 People's Republic of China; 3 Department of Cardiothoracic Surgery, The First Affiliated Hospital of Chongqing Medical University, Yuanjiagang, Chongqing, 400016 People's Republic of China

**Keywords:** Atrial fibrillation, Coronary artery bypass grafting, Cardiopulmonary bypass, High-mobility group box protein 1, Polymorphism

## Abstract

**Background:**

The inflammatory response triggered by cardiac surgery with cardiopulmonary bypass (CPB) is a primary cause of postoperative atrial fibrillation (POAF). The objective of this study was to determine the relationships between rs2249825 (C/G) polymorphism in high-mobility group box protein 1 (HMGB1) and POAF in patients who underwent coronary artery bypass grafting (CABG) under CPB.

**Methods:**

A prospective cohort study was carried out between February 2011 and January 2014. Patients who had no history of atrial fibrillation undergoing CABG with CPB were recruited in this study, and were matched based on preoperative characteristics. Blood samples were obtained before, and at 4, and 24 h after CPB. HMGB1 level was measured by enzyme immunoassay. Patients were genotyped for single nucleotide polymorphisms of HMGB1 (rs2249825). Patients were genotyped for single nucleotide polymorphisms of HMGB1 (rs2249825) using pyrosequencing method. The primary clinical end point was the incidence of POAF after surgery.

**Results:**

After matching, a total of 128 patients undergoing elective CABG with CPB were eligible for analysis. Plasma HMGB1 concentrations were increased 4 h after CPB (*p* <0.0001) and were still increased at 24 h (*p* <0.0001). The frequencies of CC, CG, GG genotypes were 21 (56.8 %), 29 (37.8 %), and 2 (5.4 %) in patients with POAF and 81.3, 16.5, and 2.2 % in patients without POAF (*p* = 0.016). CG + GG genotype was associated with high HMGB1 levels compared with the genotype CC at 4 h (*p* = 0.023), and 24 h (*p* = 0.015) after CPB. Multivariate analysis showed that age older than 60 years (OR = 1.40; 95 % CI: 1.03 to 1.89; *p* = 0.021) and allele G of polymorphisms (OR = 1.61; 95 % CI: 1.08 to 2.04; *p* = 0.034) were independent risk factors for POAF.

**Conclusions:**

The HMGB1 rs2249825 was associated with the susceptibility to POAF after CABG with CPB in a Chinese Han population.

## Background

Postoperative atrial fibrillation (POAF) is the most common complication encountered after cardiac surgery and has important clinical and economic implications [1, 2]. The incidence of POAF reported in previous studies ranging from 10 to 65 % of patients after cardiac surgery, depending on definitions and methods of detection [3–5]. Although several studies have analyzed the risk factor for POAF, the exact pathophysiology of this problem has not already been illustrated. It has been demonstrated that an increased inflammatory response correlates with the occurrence of POAF [6–8]. The complex inflammatory response to cardiac surgery using cardiopulmonary bypass (CPB) is one of the primary mechanisms in the pathogenesis of POAF. These inflammatory responses eventually leads to abnormal anisotropic conduction such as interleukin-6 (IL-6), IL-1, IL-8 and tumor necrosis factor-α (TNF-α) which results in a decrease of the conduction speed and in heterogeneous impulse propagation, which facilitates the reentry and genesis of POAF [9, 10]. Therefore, inflammation has been presumed to be implicated in its pathogenesis. However, the intensity of this inflammatory response is variable among patients and remains unpredictable. Increased evidence for heritability of the proinflammatory state suggests that individual genetic background also plays roe in modulating the magnitude of postoperative systemic inflammatory response after cardiac surgery [11].

Genetic variants, particularly single-nucleotide polymorphisms (SNPs), are critical determinants for interindividual differences in both inflammatory responses and clinical outcomes in patient with on-pump primary isolated coronary artery bypass surgery [2]. Given the broad distribution, genetic polymorphisms offer a potential explanation for the susceptibility of POAF after CABG.

High-mobility group box protein 1 (HMGB1) has been demonstrated to be as an important mediator of systemic inflammation. HMGB1 is highly conserved among mammals, showing more than 98 % of sequence identities between humans and other mammals [12]. HMGB1, as a nuclear DNA binding protein, has recently been reported to be involved in triggering sterile inflammation [13]. Extracellular HMGB-1 acts as an alarm signal to induce inflammation, proliferation and migration of immune cells [14, 15]. In addition, growing evidence has also indicated that HMGB1 plays a central pathogenic role in critical illness [15–17]. Collectively, these suggest that HMGB1 could be involved in the inflammatory responses to CABG with CPB, and may be a common risk gene for POAF after cardiac surgery.

In this study, we tested whether rs2249825 polymorphism of HMGB1 was associated with risk of POAF in a Chinese Han population.

## Methods

### Study population

This was a nonrandomized, prospective observational cohort study. To overcome bias in some baseline characteristics between patients with and without POAF, the discovery set actually were frequency-matched to the case by age (±5 years), gender, and comorbidities including diabetes, hypertension and previous myocardial infarction.

From February 2011 to January 2014, a total of 128 patients undergoing elective CABG with CPB were screened. All patients were placed on CPB. Exclusion criteria included emergency surgery, a prior history of AF, age > 80 years, left atrial volume (LAV) > 32 ml/m^2^, left ventricular ejection fraction (LVEF) < 0.30, chronic obstructive pulmonary disease (COPD), impaired renal function and current infection. This study further excluded patients with necessitating intervention to the mitral valve or POAF due to electrolyte imbalance.

All patients requiring surgical intervention received standard surgical care and postoperative intensive care unit treatment. The protocol for this study was approved by the local institutional ethics committee, and the informed consent was obtained from the patients or the patients’ next of kin.

### Surgical procedures

Standard median sternotomy was the selected surgical access to the heart for all patients and the pericardial edges were lifted. CPB was instituted using a Cobe hollow fibre membrane oxygenator and the CPB flow was regulated at 2.o to 2.4 L min^−1^ m^−2^, and moderate hypothermia (32 to 34 °C) was accomplished. The mean perfusion pressures were maintained at a range of 60 to 80 mmHg. Identical cold blood cardioplegic solution was given after cross-clamping for myocardial protection. The distal vein graft anastomoses were performed first, while the distal left internal mammary artery anastomoses to the diagonal branches and or left anterior descending were performed last. The proximal vein graft anastomosis was performed under side clamping of the aorta. After declamping, the heart was defibrillated, if needed, then the patient was rewarmed. After weaning of the CPB and decanulation the heparin was reversed with protamine sulphate.

### Blood sample collection and assay

For each patient peripheral venous blood samples were obtained through a central venous catheter at 3 different time points, which are before, and at 4 and 24 h after CPB. Samples were collected in potassium ethylenediamine tetra-acetic acid (EDTA) coated bottles, and were centrifuged at 5000 g for 5 min to remove the cellular components. The plasma obtained was stored at −80 °C for longer term storage.

The plasma samples were tested for HMGB1 levels by enzyme-linked immunosorbent assay (ELISA), using a commercially available human HMGB1 ELISA kit (USCN Life Sciences, Wuhan, Hubei, China ), according to the manufacturer’s instruction. The minimum detectable level of HMGB1 was 12 pg/ml.

### Postoperative evaluation and definition

After completion of the surgical procedure, patients were admitted to intensive care unit and when their haemodynamic and respiratory functions were stable, they were transferred to wards. All patients were monitored continuously with electrocardiographic (ECG) telemetry equipment until postoperative day 5, and their 24-h report was reviewed by a study physician or a study nurse coordinator every day for any episode of POAF. In case of clinical suspicion of arrhythmia, a standard 12-lead electrocardiogram was performed two times a day from the first postoperative day until discharge. POAF was defined as an irregular supraventricular rhythm present in the absence of P waves, which was typically sustained for more than 30 min. Therapeutic approaches for treatment of POAF included standard pharmacologic management and electric cardioversion if indicated. To avoid investigators-related biases, all the physicians involved in patients’ care were blinded to the results of the genetic analyses.

### Genomic DNA isolation and genotype analysis

Blood specimens were collected in tripotassium EDTA sterile tubes from patients after admission. Genomic DNA was extracted from whole blood using the Wizard Genomic DNA Purification Kit (Promega, Madison, WI, USA) according to the manufacturer’s protocol. The extracted DNA was stored at −20 °C until used. The primers for the SNPs rs2249825 were 5′-TGTCTGATTTTACGGAGGTTGAT-3′ (forward) and 5′-GTTTGCACAAAAAATGCATATGAT-3′ (reverse). The PCR conditions were as follows: 5 min at 94 °C followed by 35 cycles for 30 s at 94 °C, 30 s at 60 °C, and 30 s at 72 °C. The genotypes of the PCR products were determined by pyrosequencing analysis using a standard protocol as previously described [18]. Genotype was assigned as homozygous C/C, homozygous G/G or heterozygous C/G.

### Statistical analysis

Categorical variables were presented as percentages and continuous variables were expressed as mean ± SD. Categorical variables were compared by Chi-square analysis or Fisher’s Exact Test as appropriate; continuous variables were compared with Student’s t test for normally distributed values and with Mann–Whitney-Wilcoxon test for non-normally distributed variables. The Chi-square test was used to test the deviation of genotype distribution from the predicted genotype frequencies based on the Hardy-Weinberg equilibrium. Before evaluating the contribution of genetic factors, the relationship between traditional risk factors and POAF was identified using first univariate analysis followed by multiple logistic regression analysis. We selected variables for the multivariable analysis if their *p* value was 0.05 or lower in the univariable analysis and according to their clinical relevance.

The association between gene polymorphisms and incidence of POAF was measured by a 2-stage analysis approach as described in a previous study [19]. Firstly, allelic associations with incident POAF were assessed using χ2 tests. To avoid assumptions regarding the modes of inheritance, all analyses were performed using additive (homozygote major allele versus heterozygote versus homozygote minor allele), dominant (homozygote major allele versus heterozygote + homozygote minor allele), or recessive (homozygote major allele plus heterozygote versus homozygote minor allele) models for each polymorphism. Second, Odds ratios (OR) with 95 % confidence intervals (CI) were calculated by logistic regression analysis to estimate the relative risk of POAF. All statistical tests were two sided, and *p* value < 0.05 was considered statistically significant. Statistical analyses were performed using SPSS 17.0 (SPSS, Chicago, Illinois, USA).

## Results

During the study period, a total of 151 patients scheduled for CABG with CPB. Of the patients who were initially evaluated, 23 patients were excluded for having a history of AF or COPD before their operation, LAV > 32 ml/m^2^ or LVEF < 0.30, impaired renal function, death during the early days of ICU stay and CABG with valvular surgery. A total of 128 patients were finally recruited in the study, with a mean age of 65 (±10), and 82.8 % of these patients were male. Of these enrolled patients, 37 (28.9 %) presented at least one qualifying episode of POAF after cardiac surgery. Clinical and demographic characteristics of the study population are summarized in Table 1. Results of the univariate analysis showed that clinical factors associated with POAF included age (*p* = 0.002) and aortic clamp time (*p* = 0.017).

**Table 1:** Univariate analysis for patients with or without POAF

Variable	POAF (*n* = 37)	No POAF (*n* = 91)	*P* value
Age (years)	65.7 ± 6.4	59.1 ± 7.6	0.002
Gender			
Male	29 (78.4 %)	77 (84.6 %)	0.397
Female	8 (21.6 %)	14 (15.4 %)	
Diabetes	12 (32.4 %)	29 (31.9 %)	0.951
Hypertension	24 (64.9 %)	55 (60.4 %)	0.641
Hypercholesterolemia	25 (67.6 %)	59 (64.8 %)	0.768
Previous myocardial infarction	15 (40.5 %)	37 (40.7 %)	0.99
Previous cerebral attack	3 (30 %)	7 (70 %)	0.937
Peripheral vascular disease	0 (0 %)	1 (1.1 %)	0.522
Chronic renal insufficiency	1 (2.7 %)	9 (9.9 %)	0.17
NYHA score			
I	3 (8.1 %)	8 (8.8 %)	0.994
II	25 (67.6 %)	61 (67.0 %)	
III	7 (18.9 %)	18 (19.8 %)	
IV	2 (5.4 %)	4 (4.4 %)	
Diseased vessels			
1	1 (2.7 %)	2 (2.2 %)	0.967
2	4 (10.8 %)	9 (9.8 %)	
3	32 (86.5 %)	80 (88.0 %)	
LVEF ≤ 50 %	6 (16.2 %)	13 (14.3 %)	0.781
Preoperative medications			
Beta-blockers	30 (81.1 %)	78 (85.7 %)	0.513
ACEI/ARB	18 (48.6 %)	41 (45.1 %)	0.712
Ca2 + −channel blocker	19 (51.4 %)	51 (56.0 %)	0.629
Mean CPB time (min)	107.2 ± 35.4	79.1 ± 31.9	0.023
Mean aortic clamp time (min)	74.9 ± 25.1	48.6 ± 24.4	0.017
Mean intensive care unit stay (days)	5.2 ± 3.6	4.9 ± 2.7	0.81

*POAF* postoperative atrial fibrillation, *LVEF* left ventricular ejection fraction, *CPB* cardiopulmonary bypass

**Fig. 1:**
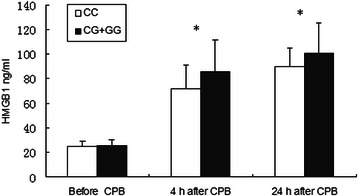
Relationship with genotype of the polymorphism in the HMGB1 gene and plasma HMGB1 concentrations before, 4, and 24 h after cardiopulmonary bypass (CPB) in patients undergoing coronary artery bypass grafting surgery. *p < 0.05

The mean plasma HMGB1 levels in these patients were 25.1 ± 4.7 ng/ml before CPB, 75.7 ± 22.3 ng/ml 4 h after CPB, and 93.1 ± 16.1 ng/ml 24 h after CPB. Patients with genotype CG + GG had significantly higher HMGB1 levels compared with those of genotype CC at 4 h (85.1 ± 26.6 ng/ml vs 71.7 ± 19.3 ng/ml [*p* = 0.023]), and 24 h (100.9 ± 24.6 ng/ml vs 89.7 ± 14.9 ng/ml [*p* = 0.015]) after CPB (Fig. 1).

Genotype frequency was 74.2 % (*n* = 95) homozygous for the C allele, 3.1 % (*n* = 4) homozygous for the G allele and 22.7 % (*n* = 29) were heterozygotes. The genotype distributions of the SNP were all consistent with the Hardy–Weinberg equilibrium (p = 0.343). Univariate analysis was performed to identify whether the rs2249825 polymorphism of HMGB1 was associated with POAF. Of those patients with POAF (*n* = 37), 21 (56.8 %) were genotype CC; 14 (37.8 %) were genotype CG; and 2 (5.4 %) were genotype GG (Table 2). Hence, allele G is associated with increased risk of POAF after cardiac surgery. To predict determinants of POAF, we included relevant clinical measurements in a multivariate logistic regression model, age, CPB time, aortic clamp time, and HMGB1 rs2249825 genotype. The multivariate analysis showed that age older than 60 years (OR = 1.40; 95 % CI: 1.03 to 1.89; *p* = 0.021) and allele G of polymorphisms (OR = 1.61; 95 % CI: 1.08 to 2.04; *p* = 0.034) were independent risk factors for POAF after cardiac surgery. POAF is associated with an increased risk of mortality and morbidity, predisposes patients to a higher risk of stroke, requires additional treatment, and increases the costs of the post-operative care.

**Table 2:** Genotype for patients with or without POAF

Polymorphism	N	No POAF, n (%)	POAF, n (%)	χ2	*P* value
Additive model					
CC	95	74 (81.3 %)	21 (56.8 %)	8.299	0.016
CG	29	15 (16.5 %)	14 (37.8 %)		
GG	4	2 (2.2 %)	2 (5.4 %)		
Dominant model					
CC	95	74 (81.3 %)	21 (56.8 %)	8.294	0.004
CG + GG	33	17 (18.7 %)	16 (43.2 %)		
Recessive model					
CC + CG	124	89 (97.8 %)	35 (94.6 %)	--	0.579*
GG	4	2 (2.2 %)	2 (5.4 %)		

*POAF* postoperative atrial fibrillation

*Fisher’s Exact Test

## Discussion

In this study, we investigated the association of SNP rs2249825 in HMGB1 gene with POAF after CABG with CPB in a Chinese Han population. The results showed that the frequency of G genotype was significantly increased in the patients with POAF, suggesting that there was a positive association of this SNP with POAF.

POAF is one of the most common complication after open cardiac surgery. Incidence of POAF after isolated CABG is lower than that of valvular cardiac surgery but is still estimated to affect approximately one-third of the total patients [20, 21]. This number is estimated to rise in a more aged patient population, as there is approximately 24 % increase in frequency of POAF with each additional 5 years of age [22]. Previous studies showed that POAF is significantly associated with increased long-term risk of mortality independent of patient preoperative severity. The risk of long-term mortality in patients that developed POAF was 29 % higher than in patients without it [23]. Although, early POAF is commonly considered relatively easy to treat and is believed to have little effect on patients’ outcomes, further research in the area of the prevention and management of POAF after cardiac surgery is needed [24, 25].

Evidence has suggested that atrial alteration of atrial conduction properties in the right atrium by inflammation after cardiac surgery is the pathologic cause of POAF [9, 26].

The acute systemic inflammatory reaction due to CPB and generalized surgical trauma is a main determinant of new-onset POAF and the modulation of inflammation will probably represent a major therapeutic goal in the short term and a promising pathway [27]. Chung et al. [7] study have shown that inflammatory markers, although generally increased after CPB, are particularly high in those patients with POAF. In addition it has been reported that interleukin-6 promoter variant appears to be implicated in inflammatory response to surgery and development of POAF [2]. Collectively, these data suggest that genetic factors may play a role in the pathogenesis of POAF after cardiac surgery. In view of the fact that inflammatory state after cardiac surgery with CPB might in fact alter the underlying atrial electrophysiology, we tested whether POAF was associated with the plasma level of HMGB1 in a Chinese Han population.

HMGB1 is a late-acting proinflammatory mediator, identified by Wang and colleagues about 15 years ago. This research led to the recognition that certain endogenous molecules, which can be passively released by stressed or necrotic cells or, in some cases, actively secreted by immunostimulated macrophages and certain other cell types, are capable of activating the innate immune system and initiating or propagating inflammation [28]. It has been reported that HMGB1 is associated with systemic inflammation and remote organ dysfunction resulting from sterile trauma, including bilateral femur fracture, ischemia-reperfusion injury, and hemorrhagic shock [29]. Proinflammatory activation signals induced by injury trigger an active release of HMGB1 from activated monocytes and macrophages; and it is also released passively by necrotic and damaged cells [14, 30]. HMGB1 released into the extracellular milieu act as a mediator to further trigger the secretion of other cytokines, such as tumor necrosis factor, IL-1, and IL-6, by macrophages and other cell types [28]. Hence, extracellular HMGB1 functions as a danger signal to responsive cells, amplifies the signal by increasing production and secretion of other proinflammatory cytokines and finally induces systemic inflammation [31].

A total of 7 SNPs have been identified to date within the human HMGB1 gene as shown by the public SNP database (www.hapmap.org), but little has been known about the biologic importance of these polymorphisms. Although Kornblit et al. [32] identified 6 polymorphisms within the entire HMGB1 gene by DNA sequence analysis of 103 healthy Caucasian Danish blood donors, Only rs1060348 polymorphism was shown to be significantly associated with outcome of patients with systemic inflammatory response syndrome in the ICU. Zeng et al. [33] selected 3 tag SNPs for the entire HMGB1 gene, and only the rs2249825 polymorphism was significantly associated with lipopolysaccharide–induced HMGB1 production by peripheral leukocytes, showing how the rs2249825 polymorphism determines HMGB1 levels.

Previous studies have identified a very interesting SNP candidate. In a study by Chew et al. [34], the polymorphism of apolipoprotein E gene, which was known to be involved in mediating inflammatory and tissue repair reactions, was found to be associated with postoperative renal dysfunction in cardiac surgery patients, suggesting the possibility of a genetic modulation of postoperative clinical outcome. Other study further showed evident correlation between the polymorphism of Interleukin-6 gene promoter of the and the development of POAF, strong augmenting in favor of an inflammatory component in the development of POAF after cardiac surgery [2]. Our results showed that the frequency of CG or CG + GG genotype was significantly increased in patients with POAF, indicating genetic predisposition play a role in this common complication after cardiac surgery.

## Conclusion

In conclusion, this study suggests that the CG/GG genotype of HMGB1 rs2249825 might cause susceptibility to POAF after cardiac surgery in a Chinese Han population. The mechanisms by which rs2249825 of HMGB1 exerts its role are not completely understood yet and further studies are needed to clarify this issue.

### Limitations

It is worthwhile to point out that there are some limitations in our study. First, as POAF is a common complication after cardiac surgery, the patients recruited from CABG might represent a subpopulation of this problem. Furthermore, the patients enrolled in this study were recruited from Chinese Han individuals and the sample size of patients included was relatively small. Thus, the results presented here need to be confirmed using different ethnic populations before to be extrapolated in the general population. Second, a further limit of our protocol lies in the fact that continuous telemetry was used only for the first 5 days after surgery and from this time on patients were submitted to surface ECG every day in case of clinical suspicion of arrhythmia. This methodology could result in the missing of some episode of transient asymptomatic atrial arrhythmia. Thirdly, we used a short follow-up period (postoperative hospitalization) following the study design of previous studies. Therefore, POAF episodes that occurred after hospital discharge were missed. Finally, and importantly, it is necessary to clarify whether the loci associated with POAF identified in the present study is only a marker or a causative variant.
